# Multidimensional Imaging of Mammary Gland Development: A Window Into Breast Form and Function

**DOI:** 10.3389/fcell.2020.00203

**Published:** 2020-03-31

**Authors:** Bethan Lloyd-Lewis

**Affiliations:** School of Cellular and Molecular Medicine, Biomedical Sciences Building, University of Bristol, Bristol, United Kingdom

**Keywords:** mammary gland development, breast cancer, 3D imaging, 4D imaging, intravital microscopy, mammary stem cells, lactation

## Abstract

An in-depth appreciation of organ form and function relies on the ability to image intact tissues across multiple scales. Difficulties associated with imaging deep within organs, however, can preclude high-resolution multidimensional imaging of live and fixed tissues. This is particularly challenging in the mammary gland, where the epithelium lies deeply encased within a stromal matrix. Recent advances in deep-tissue and live imaging methodologies are increasingly facilitating the visualization of complex cellular structures within their native environment. Alongside, refinements in optical tissue clearing and immunostaining methods are enabling 3D fluorescence imaging of whole organs at unprecedented resolutions. Collectively, these methods are illuminating the dynamic biological processes underlying tissue morphogenesis, homeostasis, and disease. This review provides a snapshot of the current and state-of-the-art multidimensional imaging techniques applied to the postnatal mammary gland, illustrating how these approaches have revealed important new insights into mammary gland ductal development and lactation. Continual evolution of multidimensional image acquisition and analysis methods will undoubtedly offer further insights into mammary gland biology that promises to shed new light on the perturbations leading to breast cancer.

## Introduction

Life is underpinned by a series of dynamic biological events tightly coordinated in space and time. Consequently, real-time visualization of cellular processes unfolding in their most relevant contexts is paramount for an in-depth understanding of tissue development and disease (Follain et al., 2017). Recent advances in rapid, high-resolution imaging methodologies, genetically-encoded fluorophores and *in vivo* models are enabling this endeavor, illuminating the dynamic cellular and subcellular events that underpin life (Follain et al., 2017). This mini-review focuses on the application of multidimensional imaging methods to the mammary gland, a secretory organ essential for mammalian offspring survival.

The adult mammary gland comprises of a branched ductal epithelium sheathed by an adipocyte-rich stroma. Two principle cell lineages form the mammary epithelial bi-layer; an inner layer of luminal cells enveloped by a layer of myoepithelial (basal) cells. Mammary gland development is a multi-stage process, occurring during embryogenesis, puberty and repeated pregnancy cycles (Watson and Khaled, 2008; Macias and Hinck, 2012). This dynamicity was first depicted in 1933 via a sequence of camera lucida drawings of murine mammary gland morphology at different stages of development (Cole, 1933). Subsequent advances in light and electron microscopy rapidly revealed the intricate architecture of the mammary epithelium, laying the groundwork for future studies into the molecular mechanisms that underlie mammary gland form and function (reviewed in Neville, 2009). The mouse is an excellent model for investigating processes regulating human mammary gland biology, providing relevant insights into the perturbations that give rise to breast cancer (Sreekumar et al., 2015).

Historically, detailed microscopic analyses of mammary gland tissues have been restricted to thin, two-dimensional (2D) sections. While informative, with enduring relevance, tissue sections lack architectural context and are hampered by assumptions regarding the uniformity of a particular 2D plane (Sale and Pavelic, 2015; Lloyd-Lewis et al., 2016). Moreover, biological entities are intrinsically three-dimensional (3D), and their true nature cannot be ascertained by a thin section (Richardson and Lichtman, 2015). Volumetric 3D imaging, therefore, is necessary to reveal the spatially complex topology of the branched mammary epithelium. In addition, as fixed tissue analyses are limited to snapshots in time, four-dimensional (4D, *x*-, *y*-, *z*-, *t-*) live cell imaging is required to interrogate the inherently dynamic processes underpinning the development and function of this complex tissue.

Herein, this mini-review provides an overview of the available strategies for high-resolution multidimensional fluorescence imaging of mammary gland tissues at the microscopic scale. Due to space constraints, technologies for imaging at the nano-, meso-, and macro-scale will not be discussed here. Subsequently, this article will briefly highlight recent 3D and 4D imaging studies that have provided important insights into mammary gland ductal development and lactation, which could not have been resolved using conventional histological techniques.

## Fluorescence Light Microscopy Platforms for High-Resolution Multidimensional Imaging

High-resolution fluorescence 3D and 4D microscopic imaging can be performed using optical sectioning techniques such as confocal (Conchello and Lichtman, 2005), multiphoton (Helmchen and Denk, 2005; Dunn and Young, 2006) and light sheet microscopy (LSFM) (Huisken et al., 2004; Keller et al., 2008). Broadly, optical sectioning acquires images of thin focal planes within thick specimens by eliminating the contribution of out-of-focus light and scatter in each image plane. This provides greater contrast, allowing stacks of images captured at serial focal planes to be computationally combined for 3D reconstruction (Conchello and Lichtman, 2005). The universal utility of these imaging approaches for multidimensional microscopy, particularly for *in vivo* cell biology, are discussed in detail elsewhere (Timpson et al., 2011; Follain et al., 2017).

In general, confocal microscopy is the most commonly used optical sectioning technique for fluorescence 3D imaging. However, confocal modalities rely on excitation wavelengths in the visible range that suffer from tissue light absorption and scattering, limiting imaging depths to superficial regions (∼100 μm) in most specimens (Conchello and Lichtman, 2005; Follain et al., 2017). Nevertheless, when important biological information can be garnered from near-surface tissue areas, confocal microscopy is associated with a number of advantages, including widespread accessibility, relatively fast acquisition speeds and flexible multicolor acquisition capabilities (Egeblad et al., 2008; Ebrahim and Weigert, 2019).

For deep tissue fluorescence imaging, multiphoton microscopes equipped with pulsed infrared lasers are frequently used. This approach relies on the simultaneous absorption of two or more low-energy infrared photons for fluorophore excitation. In turn, this confines two-photon excitation to a limited focal volume, enabling optical sectioning alongside reduced photo-toxicity and bleaching (Helmchen and Denk, 2005; Dunn and Young, 2006). Moreover, long-wavelength excitation by infrared lasers are associated with decreased tissue scattering and light absorption, facilitating deeper light penetration and imaging depths of up to 1 mm in many tissues. In addition, by exploiting the physical and auto-fluorescent properties of endogenous molecules, nonlinear multiphoton-excitation facilitate second (SHG) (Campagnola et al., 2002) or third (THG) harmonic generation imaging of non-labeled cellular components, such as collagen and lipids (Friedl et al., 2007; Weigelin et al., 2016).

Light sheet fluorescence microscopy (LSFM) is a powerful method that performs optical sectioning using a thin plane of light, allowing focal planes to be captured in a single exposure (Huisken et al., 2004; Keller et al., 2008). This facilitates rapid and long-term 3D imaging of specimens, including live mouse embryos, at high spatiotemporal resolution with minimal photodamage (Power and Huisken, 2017; Katie McDole et al., 2018; Wan et al., 2019). Similarly to confocal microscopy, however, LSFM is constrained by tissue light scattering, limiting its application to relatively transparent or thin samples (Wan et al., 2019). In addition, the unique optical geometry inherent to most current configurations pose significant barriers for sample maintenance during acquisition (Benninger and Piston, 2013), precluding *in vivo* imaging of adult mice by LSFM. Nevertheless, when combined with optical tissue clearing (discussed below), LSFM facilities rapid whole-organ 3D imaging of fixed specimens (Keller and Ahrens, 2015; Susaki and Ueda, 2016), including the mammary gland (Lloyd-Lewis et al., 2016).

## 3D Imaging Strategies for Fixed Tissues

All light microscopy methods are hampered by tissue light scattering and absorption, which ultimately defines the limit of depth penetration (Wan et al., 2019). The mammary gland is a case in point, as the adipocyte-rich stroma poses significant barriers for high-resolution, deep tissue 3D imaging. Consequently, a number of strategies are used to improve mammary gland wholemount immunostaining and depth of imaging in fixed tissues, including microdissection (Rios et al., 2014, 2016b), enzymatic digestion (Wuidart et al., 2016, 2018; Scheele et al., 2017; Lilja et al., 2018), and optical tissue clearing (Davis et al., 2016; Lloyd-Lewis et al., 2016, 2018; Elias et al., 2017; Seong et al., 2018; Chen et al., 2019; Hitchcock et al., 2019; Rios et al., 2019; Stewart et al., 2019). Tissue microdissection facilitates high-resolution 3D imaging of large areas of the ductal epithelium within stroma-divested mammary glands (Rios et al., 2014). Conversely, proteolytic digestion of mammary tissues prior to immunostaining results in improved antibody penetrations, enabling whole-gland 3D imaging of slide-mounted tissues (Wuidart et al., 2016, 2018; Scheele et al., 2017; Lilja et al., 2018). This approach, however, risks damaging or depleting epithelial and stromal cell populations within the mammary fat pad (Rios et al., 2016a), prohibiting its widespread utility. Alternatively, tissue clearing techniques can be harnessed to improve optical access and depth of imaging in intact mammary gland tissues (Richardson and Lichtman, 2015; Lloyd-Lewis et al., 2016).

Recent innovations in optical sectioning microscopy, particularly LSFM, have precipitated the development of numerous optical tissue clearing techniques aimed at rendering biological specimens transparent (Richardson and Lichtman, 2015; Tainaka et al., 2016). These methods seek to increase tissue imaging depths by minimizing light scattering caused by mismatches in refractive indices (RIs) between heterogeneous cellular components. Broadly, optical clearing methods rely on organic solvent-based (e.g., 3DISCO; Erturk et al., 2012) or aqueous reagent-based clearing agents (e.g., Scale, Hama et al., 2015; SeeDB, Ke et al., 2013; CUBIC, Susaki et al., 2014; FRUIT, Hou et al., 2015; C_*e*_3D, Li et al., 2017; UbasM, Chen et al., 2017) to equilibrate RIs within a tissue (Table 1 and recently reviewed in Matryba et al., 2019). Samples may also be hydrogel-embedded prior to clearing to preserve cellular structures (e.g., “active” and “passive” CLARITY methods; Chung and Deisseroth, 2013; Yang et al., 2014).

**TABLE 1:** An overview of the tissue clearing methods applied to mammary gland tissues and/or tumors.

							Preservation			
							
				Time to	Clearing				Mammary gland	Original
Method	Method overview	Key components	RI	clear^b^	capability	IHC	Structure	FP	references	references
3DISCO	Organic solvent based	Dichloromethane/dibenzyl ether	1.56	2 days	Strong	Difficult (iDisco)^c^	Compromised – shrinkage	Rapid loss	Erturk et al., 2012; Lloyd-Lewis et al., 2016	Erturk et al., 2012
CLARITY	Aqueous solution based – hydrogel embedding	SDS/acrylamide/Rapiclear/80% glycerol	1.52	10 days	Strong	Compatible	Preserved	Preserved	Chen et al., 2019	Chung and Deisseroth, 2013
PACT^a^	Aqueous solution based – hydrogel embedding	SDS/acrylamide/sRIMS/Rapiclear	1.45–1.46	10–14 days	Weak	Compatible	Preserved – mild expansion	Preserved	Lloyd-Lewis et al., 2016	Yang et al., 2014
Ce3D	Aqueous solution based – simple immersion	*N-*methylacetamide/Histodenz	1.49–1.5	2 h	Strong	Not tested	Not analyzed	Not tested	Rios et al., 2019	Li et al., 2017
SeeDB	Aqueous solution based – simple immersion	Fructose/thioglycerol	1.49	5 days	Moderate	Compatible	Preserved – mild shrinkage	Preserved	Davis et al., 2016; Lloyd-Lewis et al., 2016, 2018; Elias et al., 2017	Ke et al., 2013
FRUIT	Aqueous solution based – simple immersion	Fructose/Urea	1.49–1.5	3 days	Poor	Not tested	Not analyzed	Not tested	Rios et al., 2019	Hou et al., 2015
ScaleS	Aqueous solution based – simple immersion	Urea/Sorbitol	1.38	3 days	Strong	Not tested	Not analyzed	Not tested	Rios et al., 2019	Hama et al., 2015
FUnGI	Aqueous solution based – simple immersion	Urea/fructose/glycerol	1.46	2 h	Strong	Compatible	Preserved	Preserved	Rios et al., 2019	Rios et al., 2019
UbasM	Aqueous solution based – simple immersion	Urea/Amino-sugars	Not provided	7–12 days	Not shown	Not tested	Not analyzed	Preserved	Chen et al., 2017	Chen et al., 2017
CUBIC	Aqueous solution based – simple immersion	Urea/sucrose	1.48–1.49	5 days	Strong	Semi-compatible^d^	Preserved – mild expansion	Some loss^d^	Davis et al., 2016; Lloyd-Lewis et al., 2016, 2018; Seong et al., 2018; Hitchcock et al., 2019; Stewart et al., 2019	Susaki et al., 2014

*^*a*^PACT (passive clarity technique) performed using either Rapiclear or sRIMS (sorbitol RI-matching solution) for imaging in Lloyd-Lewis et al. (2016). ^*b*^Including fixation time. ^*c*^3DISCO protocol combined with optimized whole-mount immunolabeling procedures (iDISCO). Fluorescence signal is rapidly quenched using benzyl alcohol benzyl benzoate (BABB) and specialized imaging chambers are required for imaging in dibenzyl ether. ^*d*^May be improved using second generation CUBIC protocol (R1A, unpublished, protocol available at http://cubic.riken.jp/) and newer derivatives (Tainaka et al., 2018). RI, refractive index; FP, fluorescent protein; IHC, immunohistochemical analysis. Not analyzed/tested means not assessed in mammary gland tissues.*

**FIGURE 1:**
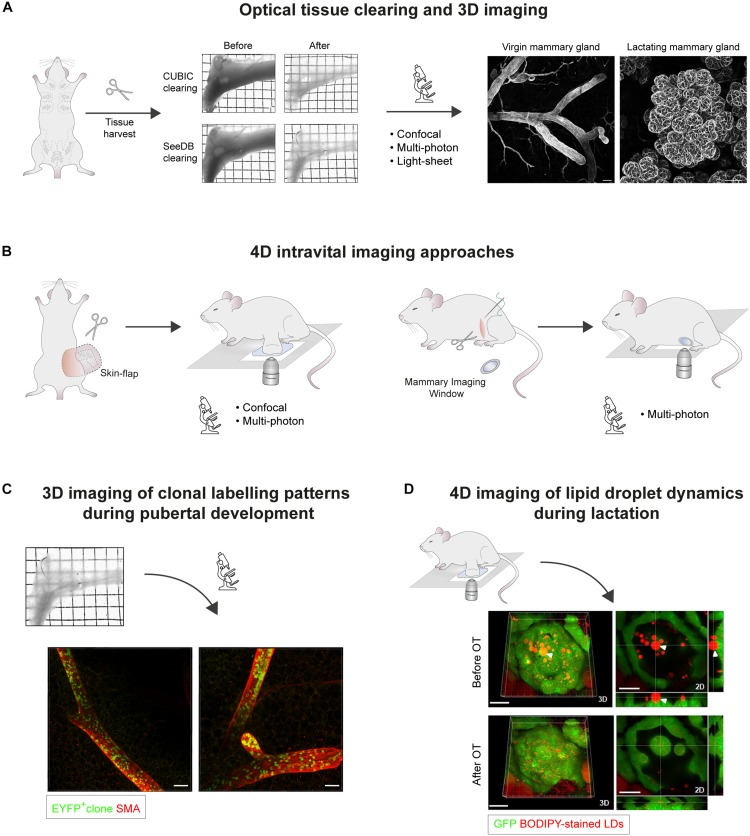
Microscopic 3D and 4D imaging of mammary gland ductal development and lactation. **(A)** Optical tissue clearing and 3D imaging of fixed mammary tissues. Transmission images of harvested abdominal mammary glands before and after tissue clearing using CUBIC or SeeDB protocols. Grid width: 2 mm. 3D confocal imaging of mammary epithelial structures immunostained for Smooth Muscle Actin (SMA) in cleared virgin and lactating mammary tissues. Scale bars, 100 μm. **(B)** 4D intravital imaging approaches. Intravital microscopy can be performed either by surgically exposing the tissue via a skin-flap incision for multiple hours (non-recovery imaging, <40 h), or by implanting optical imaging windows for longitudinal imaging spanning multiple days to weeks. While confocal microscopy is suitable for imaging superficial tissue regions, multiphoton excitation is required for deep-tissue imaging, particularly through mammary imaging windows. **(C)** Clonal patterns arising from the genetic labeling of a single EYFP+ epithelial cell in the mammary gland of a ∼7 week old *R26*^[CA]30EYFP^ mouse. SeeDB tissue clearing and immunostaining were performed prior to 3D imaging by confocal microscopy. Labeled progeny span multiple ducts and branches, and exhibit a sporadic, interspersed labeling pattern, emphasizing the importance of performing whole-gland and/or deep tissue 3D imaging for accurate clonal analysis. These patterns likely arise from the proliferation and intermixing of both labeled and unlabeled terminal end bud (TEB)-resident precursors, which have equipotent potential to contribute to ductal elongation. Scale bars, 100 μm. **(D)** Confocal intravital imaging of fluorescent BODIPY-stained lipid droplets (LDs) in surgically-exposed lactating mammary glands. Release of LDs from the apical surface is mediated by oxytocin (OT)-induced myoepithelial cell contractions. 3D images and 2D sections of the same alveolus before and after OT exposure are shown. White arrowhead points to an LD that was embedded in the cytoplasm prior to alveolus contraction. Scale bars, 30 μm. Images in **(A,C)** adapted from Davis et al. (2016) Nature Communications, under https://creativecommons.org/licenses/by/4.0/. Images in **(D)** adapted from Masedunskas et al. (2017) Mol Biol Cell, under https://creativecommons.org/licenses/by-nc-sa/3.0/.

By testing a number of these techniques in the mammary gland, a recent study demonstrated that SeeDB (Ke et al., 2013) and CUBIC (Susaki et al., 2014) protocols enable high-resolution 3D imaging of expansive regions of the mammary epithelium within its native stroma (Figure 1A and Table 1; Lloyd-Lewis et al., 2016). These protocols have subsequently been further developed (Ke et al., 2016; Tainaka et al., 2018), although they remain to be tested in mammary tissues. A recent study also determined the compatibility of CLARITY tissue clearing with 3D imaging of human breast tumor biopsies and archived paraffin embedded samples, highlighting the utility of this approach for enhanced visualization of intra-tumoral heterogeneity in breast cancers (Chen et al., 2019). Thus, optical tissue clearing and 3D imaging of surgically-resected breast tumors holds great potential for improved tumor classification, and thereby treatment strategies, in breast cancer patients. Nonetheless, several tissue clearing methods are disadvantaged by long incubation times, particularly when combined with immunostaining protocols (Richardson and Lichtman, 2015). Difficulties associated with sample mounting, in addition to antibody penetration and performance, also pose challenges for comprehensive deep tissue 3D imaging of mammary gland wholemounts and tumors (Lloyd-Lewis et al., 2016). To address these constraints, a recent study developed a new aqueous-reagent-based tissue clearing reagent (FUnGI) that renders human and murine mammary tissues transparent in 2 h (Rios et al., 2019). When combined with immunolabeling, this protocol spans 3 days, achieving uniform antibody staining that enables large-scale 3D imaging of the mammary epithelium and tumors at single-cell resolution (Rios et al., 2019). The continual development of tissue clearing reagents and 3D image analysis pipelines adapted for human organs (Zhao et al., 2020) will undoubtedly help facilitate the transfer of high-resolution 3D imaging to clinical practice.

Thus far, high-resolution deep tissue and/or whole-gland 3D imaging has mostly been harnessed in genetic fate-mapping studies in the mammary gland (Rios et al., 2014; Davis et al., 2016; Wuidart et al., 2016, 2018; Elias et al., 2017; Lilja et al., 2018; Lloyd-Lewis et al., 2018; Seong et al., 2018) and tumors (Van Keymeulen et al., 2015; Rios et al., 2019), where the ability to visualize expansive regions of the mammary epithelium is paramount for accurate and quantitative clonal analysis. Notably, in contrast to enzymatic digestion or mechanical dissection, most optical tissue clearing protocols preserve tissue and matrix architecture (Lloyd-Lewis et al., 2016). This provides opportunities, therefore, to explore interactions between mammary epithelial cells and their surrounding cellular and non-cellular [e.g., extracellular matrix (ECM)] niche by deep tissue 3D imaging (Inman et al., 2015). In this vein, two recent studies used optical tissue clearing and deep tissue 3D imaging to characterize mammary resident CD45+ leucocyte (Hitchcock et al., 2019), and more specifically macrophage (Stewart et al., 2019), populations at different stages of mammary gland development. Whilst CD45+ cells/macrophages were observed at all developmental stages, their prevalence, morphology, localization and interactions with the mammary epithelial bilayer exhibited stage-specific differences (Hitchcock et al., 2019; Stewart et al., 2019). These interesting findings suggest a surprisingly dynamic interplay between immune cells and the mammary epithelium, which could not have been revealed using conventional histological techniques.

## 4D Intravital Imaging in the Mammary Gland: Technical Considerations

*In vivo* imaging is an indispensable tool in basic, pre-clinical and clinical research, and is routinely used in medical practice (Condeelis and Weissleder, 2010). While low-resolution imaging approaches (including computed tomography, magnetic resonance imaging, and positron emission tomography) provide valuable anatomic and physiological information into biological tissues and tumors, these imaging modalities lack the resolution to visualize individual cells *in vivo*. By contrast, high-resolution intravital microscopy (IVM) facilitates real-time microscopic imaging of individual cells within intact tissues in live animals (Pittet and Weissleder, 2011). This powerful approach is increasingly harnessed in experimental and pre-clinical studies in fields spanning developmental biology, immunology, neuroscience, and cancer research (Condeelis and Weissleder, 2010; Nobis et al., 2018). Although currently limited, the utility of high-resolution IVM for clinical use (e.g., in dermatology, laser endomicroscopy) is an active area of research (Coste et al., 2019).

To undertake high-resolution IVM of internal organs, they must be made available to the microscope’s objective. The superficial location of the mammary gland makes it amenable to IVM via a “skin-flap” incision, which exposes the tissue for imaging while maintaining its structure and perfusion in the anesthetized mouse (Figure 1B; Ewald et al., 2011a, c). This strategy is appropriate for short-to-medium-term IVM of mammary glands for up to 40 h under non-recovery anesthesia (Egeblad et al., 2008; Ewald et al., 2011b, c). For consecutive IVM in longitudinal studies, however, surgical implantation of an optical mammary imaging window is required (Figure 1B; Kedrin et al., 2008; Gligorijevic et al., 2009; Ritsma et al., 2012; Zomer et al., 2013). This facilitates tracking of individual cells in live tissues over extended periods of time in near physiological conditions (Alieva et al., 2014). Cell type-specific fluorescent reporters, optogenetic tools and dyes can be combined for simultaneous imaging by multi-color IVM, allowing dynamic interactions between different mammary cell types and cellular structures to be visualized *in situ* (Ellenbroek and Van Rheenen, 2014; Nobis et al., 2018; Perrin et al., 2019). The majority of IVM studies rely on multiphoton modalities for deep tissue imaging (Pittet and Weissleder, 2011; Ellenbroek and Van Rheenen, 2014; Perrin et al., 2019). Nevertheless, the increased surface epithelial mass and lower adipocyte content of lactating mammary tissues and tumors, for example, make these contexts more acquiescent to confocal IVM (Ebrahim and Weigert, 2019).

For visualizing biological phenomenon that remain beyond the capabilities of current IVM tools, alternative *ex vivo* approaches may be used. For example, limited 4D imaging can be performed on excised mammary gland tissue pieces (Davis et al., 2015). Inadequate diffusion of extracellular molecules into thick adult tissues, however, results in artifacts such as tissue hypoxia, restricting this approach to short-term imaging (Shamir and Ewald, 2014; Davis et al., 2015; Lloyd-Lewis et al., 2017). Conversely, many fetal tissues, including the embryonic mammary gland, are able to be maintained *ex vivo* in explant cultures for extended periods (Kratochwil, 1969; Hens et al., 2007; Voutilainen et al., 2012, 2013). Embryonic mammary buds and their surrounding mesenchyme can be established in culture from embryonic day E11.5, allowing real-time *ex vivo* visualization of mammary embryonic branching morphogenesis (Voutilainen et al., 2012, 2013). Mammary embryonic explant cultures, therefore, represent a powerful and accessible tool for dissecting the cellular mechanisms underlying embryonic mammogenesis, an often overlooked phase in mammary gland development. Alternatively, 3D *in vitro* mammary cell culture systems – including mammary organoids that recapitulate the organization and epithelial hierarchy observed *in vivo –* can be used for real-time imaging of mammary epithelial cell behaviors in an experimentally tractable setting (Simian et al., 2001; Debnath et al., 2003; Fata et al., 2007; Ewald et al., 2008; Pasic et al., 2011; Jardé et al., 2016). As this mini-review is focused on imaging mammary gland tissues, these systems will not be discussed further here (for further details see; Shamir and Ewald, 2014; Rios and Clevers, 2018).

## Multidimensional Insights Into Mammary Gland Development

3D and 4D imaging of the mammary gland is increasingly used to address fundamental questions relating to breast biology and cancer. The *in vivo* accessibility of this tissue makes it a particularly excellent model system for high-resolution intravital imaging of tumorigenic processes. The application of IVM to study tumorigenesis, including mammary, has been extensively reviewed elsewhere (Condeelis and Weissleder, 2010; Ellenbroek and Van Rheenen, 2014; Suijkerbuijk and van Rheenen, 2017; Nobis et al., 2018). The following, instead, highlights recent IVM and 3D imaging studies focused on physiological mammary gland development and function, and the insights revealed using these approaches.

### Multidimensional Imaging of Mammary Ductal Morphogenesis

While the mammary epithelium begins its morphogenetic journey in the embryo, the majority of its development occurs postnatally. Hormonal stimulation during puberty promotes the elongation and branching of a rudimentary ductal tree, fueled by the proliferative activity of adult mammary stem/progenitor cells housed in terminal end bud (TEB) structures (Watson and Khaled, 2008; Macias and Hinck, 2012). The differentiation potential of these cells – i.e., their ability to generate one or both of the mammary epithelial cell lineages – is an area of intense interest. Early population-based genetic fate-mapping studies in the postnatal mammary gland generated conflicting results, providing evidence in support of both unipotent and bi/multipotent capacities of adult stem/progenitor cells under physiological conditions (for a detailed overview see Lloyd-Lewis et al., 2017; Seldin et al., 2017; Rodilla and Fre, 2018). Discrepancies between these studies may be, in part, attributable to the temporal and promiscuous labeling of cells by selected pathway-specific or lineage-specific promoters. Misleading results may also have arisen due to the limited power of population-based lineage tracing to accurately detect single clones using 2D mammary tissue sections, particularly when labeling is performed above clonal density (Lloyd-Lewis et al., 2017).

To resolve these inconsistencies, more recent genetic fate-mapping studies in the mammary gland – encompassing single cell, neutral, or saturation lineage tracing techniques – have relied on deep tissue and/or whole-gland 3D imaging for quantitative clonal analyses. By combining fate-mapping techniques with the 3D imaging strategies described above, it was established that unipotent luminal and basal progenitors maintain the mammary epithelial lineages during postnatal mammary gland development (Davis et al., 2016; Wuidart et al., 2016; Scheele et al., 2017; Lloyd-Lewis et al., 2018). Moreover, 3D imaging revealed that the progeny of a single labeled cell can be distributed in a stochastic, interspersed pattern throughout the length of the branching epithelium (Figure 1C). These studies indicate that, despite displaying heterogeneity in gene expression at the single cell level (Scheele et al., 2017), proliferative, unipotent TEB-resident cells actively and stochastically contribute to mammary ductal development (Davis et al., 2016; Lloyd-Lewis et al., 2017, 2018; Scheele et al., 2017). Static lineage tracing methods, however, are limited in their ability to reveal the dynamics of individual clone behaviors, necessitating the use of IVM in this context (Scheele et al., 2017; Fumagalli et al., 2019). Interestingly, time-lapse IVM of mammary gland ductal development revealed that TEB-resident mammary epithelial cells continually divide and intermix, with each lineage-restricted cell type maintaining equipotent potential to contribute to ductal elongation (Scheele et al., 2017; Fumagalli et al., 2019). Notably, these quantitative 3D and 4D imaging methods provide avenues for biostatistical modeling of mammary stem/progenitor cell fate, and how this translates into organ structure (Paine et al., 2016; Wuidart et al., 2016; Scheele et al., 2017; Lilja et al., 2018). Thus, when combined with genetic lineage-tracing, the ability to image the mammary epithelium in multiple dimensions (Davis et al., 2016; Wuidart et al., 2016, 2018; Scheele et al., 2017; Lilja et al., 2018; Lloyd-Lewis et al., 2018) has provided important insights into clonal dynamics and cell behaviors during mammary gland development that could not have been attained by examining thin tissue sections (Sale and Pavelic, 2015; Lloyd-Lewis et al., 2017).

While recent genetic fate-mapping studies have demonstrated the unipotency of postnatal mammary lineage precursors in physiological conditions, the durable plasticity of these cells is becoming increasingly apparent (Seldin et al., 2017; Wahl and Spike, 2017; Rodilla and Fre, 2018). Unipotent precursors have been shown to reacquire multi-lineage differentiation capacity in transplantation assays (Stingl et al., 2006; Van Keymeulen et al., 2011; van Amerongen et al., 2012), in response to oncogenic induction (Liu et al., 2007; Koren et al., 2015; Van Keymeulen et al., 2015; Tao et al., 2017) and upon ectopic expression of critical fate determinants of the opposing lineage (Lilja et al., 2018; Wuidart et al., 2018). A recent study also demonstrated that genotoxic exposure results in mammary epithelial cell hyperplasia and lineage infidelity, possibly mediated by signals from the tissue microenvironment (Seldin and Macara, 2019). The future application of IVM in this context is fundamental for revealing the dynamic cellular processes and behaviors underlying mammary epithelial cell plasticity (Fumagalli et al., 2019). Moreover, as this plasticity is likely exploited during mammary tumorigenesis (Liu et al., 2007; Koren et al., 2015; Van Keymeulen et al., 2015; Hein et al., 2016; Tao et al., 2017) – possibly via reactivation of embryonic developmental programs in adult breast tissues (Spike et al., 2012; Zvelebil et al., 2013; Rodilla and Fre, 2018) – an improved understanding will provide important insights into the critical steps leading to breast cancer initiation.

Mammary ductal morphogenesis is heavily dependent on reciprocal interactions between epithelial cells and the microenvironment (Inman et al., 2015; Lloyd-Lewis et al., 2019). Mammary tissue resident macrophages, for example, are recruited to TEB structures during puberty, and have been shown to be essential for normal ductal development (Gouon-Evans et al., 2000, 2002). Preliminary IVM studies in pubertal *Csf1r-EGFP* macrophage reporter mice (Sasmono et al., 2003) revealed that macrophages adjacent to putative TEB structures move rapidly along collagen fibrils, where they promote collagen fibrillogenesis to steer TEB invasion through the mammary fat pad (Ingman et al., 2006). Interestingly, recent 3D deep tissue imaging in optically-cleared mammary tissues revealed that macrophages envelop and infiltrate TEB structures (Stewart et al., 2019), and can intercalate between the epithelial bilayer within ductal regions (Hitchcock et al., 2019; Stewart et al., 2019). Collectively, these 3D and 4D imaging studies suggest a close functional relationship between macrophages and the mammary epithelium, supporting recent findings that established macrophages as important components of the mammary basal stem/progenitor cell niche (Chakrabarti et al., 2018). Detailed insights into these intriguing results awaits further IVM studies of mammary ductal development in *Csf1r-EGFP* mice (Stewart et al., 2019).

### Multidimensional Imaging of the Lactating Mammary Gland

Pregnancy is marked by a distinct phase of mammary epithelial growth, branching, and differentiation, resulting in the formation of abundant secretory (milk-producing) lobuloalveolar structures (Watson and Khaled, 2008). Milk secreted into the alveolar lumen is expelled for the suckling neonate by the contraction of alveolar basal cells in response to maternally-produced oxytocin, a process dependent on calcium ions (Gimpl and Fahrenholz, 2001; Haaksma et al., 2011; Davis et al., 2015). Lipids, particularly triacylglycerols, are major milk constituents (Oftedal, 1984) that are packaged and secreted in the form of membrane-coated lipid droplets (LDs) during lactation (Walther et al., 2017). While classical biochemical and morphological analyses have revealed valuable insights into LD assembly, fusion and secretion, the kinetics underlying this dynamic process remained unclear (Mather and Keenan, 1998; McManaman, 2012).

To address this, a recent study performed time-lapse IVM of fluorescent BODIPY-stained LDs in lactating mammary glands to measure their dynamics at peak lactation (Masedunskas et al., 2017). This approach showed that LDs transit to the cell apex by relatively slow and intermitted rates of directed motion (∼0–2 μm/min) and that, regardless of size, fusion of pre-existing LD underlined their growth. Notably, it was observed that oxytocin-induced myoepithelial cell contraction is required to release mature LDs from secretory cells into luminal spaces (Figure 1D; Masedunskas et al., 2017). This suggests that LD droplet secretion is intermittently stimulated by milk let-down (Masedunskas et al., 2017), and is not a continuous process as previously assumed from static observations (Mather and Keenan, 1998; Neville, 2009; McManaman, 2012). Intriguingly, alveolar cells switch their cellular function from LD secretion to uptake during mammary gland involution, triggering a complex program of cell death that returns the mammary gland to a near pre-pregnant state (Kreuzaler et al., 2011; Sargeant et al., 2014). Although fraught with difficulties, IVM studies investigating the mechanisms and dynamics of LD uptake during involution is an aim for the future.

Seeking to assess the spatiotemporal dynamics of oxytocin-induced alveolar contractions, a recent study performed 4D *ex vivo* imaging of mammary tissue pieces from lactating mice engineered to express a Ca^2+^ fluorescent indicator in myoepithelial cells (Stevenson et al., 2019). This approach revealed that Ca^2+^ oscillations couple to myoepithelial cell contractions, which physically deform the inner luminal cell layer for milk ejection (Stevenson et al., 2019). Interestingly, 4D *ex vivo* imaging showed that Ca^2+^-contraction coupling similarly occurs in ductal myoepithelial cells, indicating that they actively participate in milk ejection during lactation (Stevenson et al., 2019). Together, these recent 4D *in vivo* and *ex vivo* imaging studies (Masedunskas et al., 2017; Stevenson et al., 2019) have provided valuable insights into the dynamic mechanisms underlying milk lipid production, secretion, and expulsion during lactation, building on findings obtained using static measures (Mather and Keenan, 1998; Gimpl and Fahrenholz, 2001; Neville, 2009).

The benefits of 3D imaging over conventional 2D histological techniques is particularly evident when imaging densely packed tissues such as the lactating mammary gland (Rios et al., 2016b). For example, while binucleated secretory luminal cells are readily discernible by 3D imaging (Rios et al., 2016b; Hitchcock et al., 2019) their prevalence is likely underestimated when analyzing mammary tissue sections (Oliver et al., 2012; Hughes and Watson, 2018). The impact of polyploidy – a consequence of the requirement for DNA synthesis for lactation (Banerjee et al., 1971; Banerjee and Wagner, 1972; Smith, 2016) – on LD frequency and dynamics, however, remains unclear. Moreover, recent 3D imaging of optically-cleared lactating tissues revealed that macrophages closely mirror the stellate morphology of adjacent and contacting alveolar myoepithelial cells, a phenotype that is indistinguishable in thin tissue sections (Hitchcock et al., 2019; Stewart et al., 2019). The functional significance of this behavior, however, remains to be elucidated (Hitchcock et al., 2019; Stewart et al., 2019).

## Concluding Remarks

Tissue development and function depend on highly co-ordinated programs of cell proliferation, differentiation, migration, communication, and death. Static 2D measurements alone are insufficient to unravel this complexity. Deep tissue 3D imaging approaches are providing avenues to obtain detailed, spatially integrated insights into the inner workings of the mammary gland, and possess great potential for improving breast tumor classification and characterization in future clinical practice. In addition, the advent of high resolution IVM is transforming the ability to explore the dynamic cellular behaviors governing tissue physiology and dysfunction in near native contexts. High-resolution IVM is increasingly harnessed in experimental and translational breast cancer research, providing valuable dynamic information into mammary tumor growth, progression, metastasis and therapeutic response that ultimately may impact patient care (Condeelis and Weissleder, 2010; Ellenbroek and Van Rheenen, 2014; Suijkerbuijk and van Rheenen, 2017). In contrast, the application of IVM to study mammary gland postnatal development is lagging. Indeed, the light-scattering adipose stroma that shrouds the mammary epithelial tree poses significant challenges for high-resolution *in vivo* imaging of normal and pre-neoplastic ductal structures. Nevertheless, continual improvements in imaging tools, including multiphoton lasers (Andresen et al., 2009), adaptive optics (Rueckel et al., 2006), sensitive detectors and image processing methods (Gligorijevic et al., 2014; Weigert et al., 2018; Perrin et al., 2019) hold great promise for future IVM studies into mammary gland development. The burgeoning application of the multidimensional imaging approaches described herein to the mammary gland will undoubtedly herald a new era in our investigation and understanding of breast biology.

## Author Contributions

BL-L conceived and wrote the entire manuscript.

## Conflict of Interest

The author declares that the research was conducted in the absence of any commercial or financial relationships that could be construed as a potential conflict of interest.
